# Malaria in rural Burkina Faso: local illness concepts, patterns of traditional treatment and influence on health-seeking behaviour

**DOI:** 10.1186/1475-2875-6-106

**Published:** 2007-08-08

**Authors:** Claudia Beiersmann, Aboubakary Sanou, Evelyn Wladarsch, Manuela De Allegri, Bocar Kouyaté, Olaf Müller

**Affiliations:** 1 Department of Tropical Hygiene and Public Health, Medical School, Ruprecht Karls Universität Heidelberg, INF 324, 69120 Heidelberg, Germany; 2 Centre de Recherche en Santé de Nouna (CRSN), BP 02, Nouna, Burkina Faso, Africa; 3 Institute of Social Anthropology, Ruprecht Karls Universität Heidelberg, Sandgasse 7, 69117 Heidelberg, Germany; 4 Centre National de Recherche et de Formation en Paludisme, Ouagadougou, Burkina Faso, Africa

## Abstract

**Background:**

The literature on health care seeking behaviour in sub-Saharan Africa for children suffering from malaria is quite extensive. This literature, however, is predominately quantitative and, inevitably, fails to explore how the local concepts of illness may affect people's choices. Understanding local concepts of illness and their influence on health care-seeking behaviour can complement existing knowledge and lead to the development of more effective malaria control interventions.

**Methods:**

In a rural area of Burkina Faso, four local concepts of illness resembling the biomedical picture of malaria were described according to symptoms, aetiology, and treatment. Data were collected through eight focus group discussions, 17 semi-structured interviews with key informants, and through the analysis of 100 verbal autopsy questionnaires of children under-five diagnosed with malaria.

**Results:**

*Sumaya, dusukun yelema, kono*, and *djoliban *were identified as the four main local illness concepts resembling respectively uncomplicated malaria, respiratory distress syndrome, cerebral malaria, and severe anaemia. The local disease categorization was found to affect both treatment and provider choice. While *sumaya *is usually treated by a mix of traditional and modern methods, *dusukun yelema *and *kono *are preferably treated by traditional healers, and *djoliban *is preferably treated in modern health facilities. Besides the conceptualization of illness, poverty was found to be another important influencing factor of health care-seeking behaviour.

**Conclusion:**

The findings complement previous evidence on health care-seeking behaviour, by showing how local concepts of illness strongly influence treatment and choice of provider. Local concepts of illness need to be considered when developing specific malaria control programmes.

## Background

At least one million malaria deaths occur each year in sub-Saharan Africa (SSA), with the great majority among young children in rural areas [1,2]. In such areas, home treatment with chloroquine, antipyretics, and traditional remedies is the most frequent response of caretakers to fever episodes in children [2,3].

The main component of malaria control in SSA is rapid diagnosis and effective treatment of clinical attacks, either at home or at modern health facilities. Success of this strategy, however, is closely linked to people's behaviour. The literature on health care-seeking behaviour has clearly shown that the mere availability of services and drugs does not guarantee that people will access such services and drugs. Factors such as the household socio-economic status, parents' education, the household head's sex and age, the distance to the health facility, and the quality of health care services have all been found to influence people's treatment and provider choices [3-10].

Most of the literature on health care-seeking behaviour, however, stems from the field of health economics and as such, it is based on quantitative analyses. Therefore, it inevitably fails to explore if and what role local concepts of illness may play in shaping treatment and provider choices. To the present day, only a handful of studies have looked at how local concepts of illness affect people's choices regarding malaria treatment in SSA [11-17], although such knowledge is essential to guide the development of effective malaria control programmes.

## Methods

### Study area

The study was conducted between December 2003 and March 2004 in seven villages of the Nouna Health District, located in northwestern Burkina Faso, approximately 300 km from the capital Ouagadougou.

A sub-portion of the Nouna Health District is under demographic surveillance by the Centre de Recherche en Santé de Nouna (CRSN), a well-established research institution. The CRSN is running a demographic surveillance system (DSS) which covers the population of the town of Nouna (25,000 people) and that of 41 surrounding villages (35,000 people) [18]. The DSS includes verbal autopsy diagnoses of all deaths in the study area. The families of the deceased persons are visited by trained interviewers one to three months after the death and a standardized questionnaire is filled in, which collects information on the signs and symptoms the patient presented before his/her death, as well as information on health care-seeking behaviour. Based on the answers given by the family members, two independent local physicians assign a final diagnosis.

Malaria is highly endemic, but also highly seasonal in the study area. Most malaria transmission takes place during or shortly after the rainy season which usually lasts from June until October [19]. At the time of the study, modern health care services in the DSS area were limited to four village-based health centers, Centre de Santé et Promotion Social, (CSPS) and one district hospital, Centre Médical avec Antenne (CMA), in Nouna town.

The study area is extremely poor. Most of the people work as subsistent farmers, producing crops like millet and sorghum. The region is predominantly inhabited by the Dafing/Marka, Bwaba, Mossi, and Peulh ethnic groups. Besides French as the official language, the main local language is Dioula, which is spoken and understood by the majority of the population. In Burkina Faso, around 60% of the population is Muslim, 30% Christians, and 10% Animists. Traditional African beliefs still influence both monotheistic religions to a large degree.

### Study design and procedures

The study relied on a variety of qualitative methods, ranging from focus group discussions (FGD) to individual interviews to document analysis. Likewise, it relied on variety of informants ranging from mothers to health care providers, from 'guérisseurs' to traditional birth attendants. Throughout the paper, we use the term guérisseur to indicate traditional health practitioners. The term combines two different types, the diviner mediums and the herbalists. The guerisseurs' methods range from eye- and breath diagnostic to touching of the body, from the use of plant, animal, and mineral products to practicing spiritual healing.

Data were collected on four local illness concepts known from previous work in Burkina Faso [20-22] to resemble the biomedical picture of malaria with a special focus on their treatment:

• *Sumaya *(resembling the biomedical picture of uncomplicated malaria)

• *Dusukun yelema *(resembling the biomedical picture of respiratory distress syndrome, of which 80% are due to malarial acidosis [23])

• *Kono *(resembling the biomedical picture of cerebral malaria)

• *Djoliban *(resembling the biomedical picture of severe anaemia)

For simplicity reasons, the concepts are here named in Dioula, although data were also collected in the local languages Bwamu and Moré. Four villages of the Nouna DSS area were purposefully sampled [24] as they were considered to be representative of the four major ethnic groups residing in the study area (Dafing, Bwaba, Mossi, Peulh).

In each village, two FGDs were conducted: one with young (20–30 years) and one with old (40–55 years) mothers of at least one child. Mothers are indispensable informants as they are responsible for the health of their children. Women were invited to attend the FGD directly by their village leader, who had in turn been contacted and asked to act as an intermediary by a CRSN staff member. The average number of participants was 11 and the average duration of a FGD was one hour. The first and second author developed the interview guide, which focused on the four local illness concepts outlined earlier and on the relevant health care-seeking behaviour and specific treatment procedures. The FGD were moderated in one of the local languages (Dioula, Bwamu, Moré) by trained CRSN interviewers.

Additionally, 17 semi-structured interviews were held with key informants:

• nine interviews with guérisseurs (identified during FGDs) from seven villages of the CRSN study area

• four interviews with all senior nurses from the four CSPS in the study area

• four interviews (convenient sample) with traditional birth attendants (TBAs) from four villages of the study area

The first and second authors developed separate interview guides to best suit the tone to be maintained with each group. The themes addressed, however, converged towards: the guérisseurs personal background and their role within the health system; the four local illness concepts and the relevant treatment, both traditional and biomedical; the cost of treatment; and the collaboration between biomedical and traditional health institutions. The interviews were conducted by trained CRSN interviewers and the average time of an interview was an hour.

After explaining the aim of the study and the dynamics of the discussion, both the FGD moderators and the interviewers always sought the respondents' informed consent. Both the FGD and the individual interviews were tape recorded with the permission of the participants and later transcribed into French by the interviewers. The transcribed text was coded inductively and analysed by the first author, who then discussed the relevance of the findings and the policy implications with all other authors.

Individual interviews were done with the guérisseurs, traditional birth attendants and senior nurses of the CSPS who, on the basis of their special expertise, were regarded to be in the position to provide interesting information.

Finally, a document analysis was conducted by the first author on a random sample of 100 verbal autopsy questionnaires from the years 1999–2002 with the diagnosis of malaria in young children (< 5 years). The content of the questionnaires was reviewed regarding issues like personal data, illness history, and treatment seeking-behaviour before death.

Systematically comparing findings from the FGDs, from the individual interviews, and the document analysis on the VA questionnaires provided a powerful source of triangulation [24].

## Results

The presentation of the findings is organized in three different sections. The first two sections report findings from the FGD and the individual interviews and focus on describing the local illness concepts and the relevant treatment and provider choices. The last section reports findings from the analysis of the verbal autopsies. Verbatim quotations are used to illustrate findings in the first two sections. Speech reported in the article was translated into English by the first author.

### Description of the local illness concepts

All respondents (mothers, guérisseurs, TBAs) described the four local illness concepts in very similar ways.

#### Sumaya

This term literally means humidity or coolness and is known as '*illness of the cold*'. *Sumaya *is most consistent with the biomedical definition of uncomplicated malaria. It is very frequent and found among all age groups. It is considered to be a serious illness. Cited symptoms are fever, weakness, cold, loss of appetite, general body pain, diarrhoea, and vomiting. *Sumaya *is perceived to be caused by natural factors such as the dirty environment, the climate (cold, wind) or certain kinds of food (e.g. too much sugar, too much condiment). Mosquitoes are usually not seen as the cause – mentioned only by one TBA and two guérisseurs who both stressed that it is a cause only during the rainy season.

#### Dusukun yelema

This illness concept can be translated as '*displaced heart*'. It is believed that the heart leaves its usual place in the body and shifts to another part (e.g. into the leg or the back). This can be caused by either natural factors, e.g. another illness such as severe *sumaya*, severe cough or other long-term or chronic disorders, or by supernatural factors such as the action of a sorcerer/an evil person. The two most significant symptoms of *dusukun yelema *are respiratory difficulties and vomiting. It is, thus, very close to the biomedical definition of respiratory distress syndrome. Other symptoms are fever, loss of appetite, and diarrhoea. Although considered to be not very frequent, d*usukun yelema *is perceived as very dangerous and often fatal.

#### Kono

The local concept of *kono *is very close to the biomedical picture of cerebral malaria and characterized by convulsions and coma. *Kono *is an illness exclusively restricted to young children (3 months – 5 yrs.). It is considered a very serious illness which has often fatal outcomes.

"*In our community there is no child illness which is worse than kono*." (guérisseur F, Mossi)

"*Many children have died because of it*." (young mother, FGD A, Bwamu)

Literally translated *kono *means bird, so one can say that it is the *'bird-illness'*. The name is explained first by the fact that the movement of a child's arms during an attack resembles the movement of a bird's wings, and second by the belief that the cause of illness is also seen in the action of a bird. The bird provokes the illness whilst flying over a woman sleeping with her child outside during the night. If a pregnant woman sleeps outside during the night and the bird passes over her, the child may attract *kono *once born. *Sumaya *was also mentioned as a possible cause of *kono*. However, it must be stressed that the two are perceived as entirely different illnesses and not – as in biomedicine – as the mild and severe forms of the same disease, i.e. malaria.

#### Djoliban

*Djoliban *means "*the blood is finished*". This term in a biomedical sense describes a state of severe anaemia. The study population had not much knowledge about *djoliban*. According to the interviewees it is a very rare, but life-threatening illness. The main symptom of *djoliban *is that the body, eyes, and palms of the patient become pale. Further cited symptoms are fever, vomiting and loss of appetite. Causes may be a long-term or chronic illness, as well as *sumaya*, weakness, over-exertion, and insufficient food.

### Treatment of the local illness concepts

Summarized below are the results from the FGDs and individual interviews. In case of different opinions regarding treatment this is indicated.

#### Sumaya

*Sumaya *is usually treated at home, by mothers themselves, using a mixture of herbs and modern drugs such as paracetamol and chloroquine. The herbal remedy – usually plant leaves – is generally prepared as decoction (boiled in hot water) and then applied to the child through bathing and/or drinking.

Two different treatments were mentioned by the old mothers of FGD A, Bwamu: they mentioned the use of ash (although it was not explained what kind of ash or how to use it) and burning the hooves of a donkey to apply the remnant into the infant's anus. One other treatment was mentioned in FGD C, old mothers, Mossi: blowing into the child's ears will help the child recover.

#### Dusukun yelema

Mothers stated that this illness is usually treated by – in most cases female – guérisseurs performing a massage. Of the nine guérisseurs interviewed, five admitted to treat *dusukun yelema *– including both of the two female guérisseurs. They treat this condition with herbs or massages. These are often used in combination.

Nevertheless, some women treated this illness by themselves, using herbs and/or massages. In FGD A, Bwamu, a different treatment was mentioned by the young mothers: applying the ash of a burned mushroom (*parkia biglobosa*) onto the child's chest or grinding the shell of a tortoise to apply the powder onto the child's chest.

TBAs denied treating *dusukun yelema*. Two TBAs mentioned that they advice treatment-seeking mothers to visit a CSPS with their children. The other two stated that they are not visited by mothers whose children suffer from *dusukun yelema*, as guérisseurs are responsible for the cure of this illness:

"...*mothers don't visit me, but there are guérisseurs who treat it. There are some who massage the children*." (TBA A, Dafing).

#### Kono

Also with regard to *kono*, mothers and TBAs stated that the illness is treated by guérisseurs.

"*You take the child to the guérisseurs who will give you some remedies*." (TBA A, Dafing).

Seven of the interviewed guérisseurs explicitly stated that they treat *kono*. Herbal preparations are here one possible form of treatment. Often, roots are transformed into a powder which is added to water or thrown into a fire. The child has then to inhale the smoke of the fire or drink the water. The child is seldom bathed in the water. Only once was the introduction of the powder into the child's nostrils or anus mentioned. Besides these herbal treatments, guérisseurs cited several other treatment procedures, such as fabricating a feather chain (also given as protection), saying incantations or giving a massage.

The feather chains were also mentioned as a common form to heal *kono *by mothers and TBAs. Made out of the feathers of a bird, these chains are usually manufactured by the guérisseurs and may be directly purchased by them or bought on the local market and are tight around the child's neck, arm, wrist, or hip. There is a great variety of chains available, ranging from simple chains – only containing one feather – to more "luxurious" versions with several feathers, some of them stitched into small leather pockets.

One single mother (young mother, FGD C, Mossi) mentioned an additional treatment. She suggested that to treat *kono*, one needs to place the child on the ground below the edge of the house roof and to shed water down the roof onto the child to allow him to recover immediately.

Furthermore in one FGD (young mothers, Peulh), one verbal autopsy and in informal conversations it was mentioned that if a child with *kono *is brought to a health centre or hospital and receives an injection there, this would be fatal to the child.

#### Djoliban

The treatment for *djoliban *is considered to be the domain of the modern health facilities. People state that there is no indigenous ("African") remedy for it.

"*There is no African remedy for this*." (old mother, FGD B, Peulh)

"*To be honest, African people don't have this remedy*." (old mother, FGD C, Mossi)

If guérisseurs or TBAs diagnose *djoliban *they refer the patient to a modern health institution.

"Djoliban is rare, if this happens and I diagnose that it is djoliban, I refer the patient to the doctors." (Guérisseur C, Marka).

"*It is our nurses who do it. Me, I don't heal it*." (TBA D, Bwaba).

Only two or three respondents mentioned to treat *djoliban *– with plants. Two times it was added that feeding the child with specific food may also contribute to the cure.

### Further factors influencing treatment seeking behaviour

One factor influencing the choice for traditional treatment frequently mentioned in the focus group discussions was lack of money.

"*It is because of lack of money*."(old mother, FGD A, Dafing)

"*It is poverty. There are moments when your child falls sick and you don't have money at hand*." (old mother, FGD B, Peulh)

Other main reasons cited were the perceived efficacy of the treatment and habit.

"*Lack of money, it's true. But the plant leaves [traditional treatment], we are born with this, we cut plant leaves and bath the child with it*." (young mother, FGD C, Mossi)

"*We are born here with it, we find [the traditional treatment] with our parents and also it is effective, that's why we do it*." (young mother, FGD D, Bwamu)

"*This is what we have seen from our ancestors*." (young mother, FGD D, Bwamu)

#### Collaboration between the traditional and formal health sector

Guérisseurs reported that they are used to refer patients to modern health facilities if the illness is not in their sphere of responsibility or if the patient does not improve after treatment.

"*When you see that the products [the guérisseurs' traditional treatment] cannot heal, you bring him [the patient] to the hospital*." (guérisseur A, Bwamu).

"*If we are not able to treat the illness, we tell him [the patient] to visit a modern health center*." (guérisseur D, Mossi).

Besides these referrals there is no collaboration between the two sectors, although the great majority of guérisseurs clearly stated that they would like to collaborate with the modern health institutions. This would in their view contribute to reducing the disease burden in Burkina Faso.

"*If we unite to work together, that's what will bring forward the country. The illness, which exhausts us, will diminish*." (guérisseur E, Marka).

"*In fact it is the illness which has been the cause of our underdeveloppement. If someone doesn't know about a domain, someone else knows about it. If we unite we form one force*." (guérisseur I, Marka).

However, the guérisseurs made only a few suggestions regarding how to implement such collaboration. For example, they suggested working together with the health staff in the modern health facilities by contributing with their knowledge about illnesses, by treating patients with their own medicaments, or by treating patients referred to them by the modern health institutions. Furthermore, the guérisseurs strongly pointed out that they do not feel appropriately recognized and respected by the staff of health centers and hospitals.

"*If we poor people try to approach them [formal health staff], they don't respect us*." (guérisseur C, Marka)

### Analysis of the verbal autopsies

#### Characteristics of study group

Among the 100 children with a post mortem diagnosis of malaria, there were 45 females and 55 males. 38 of the deceased children had lived in Nouna town and 62 in the surrounding villages. The great majority of the children (76%) had died during the first two years of life as well as during the first week after the onset of symptoms (72%).

#### Causes of death

In the VA questionnaires the respondents were specifically asked which illness in their view had caused the childrens' death. Table 1 shows the perceived causes of death according to the four local concepts of illness described earlier. *Sumaya *and *kono *were the most frequently mentioned malaria-related categories, while *djoliban *was only mentioned once and *dusukun yelema *was not mentioned. Furthermore there was a great variety of other illnesses listed, not only in Dioula, but also in other local languages.

**Table 1:** Perceived causes of young children's deaths in verbal autopsy questionnaires with a malaria diagnosis

**Cause of death**	**Number of children**
*Sumaya*	37
*Kono*	21
*Djoliban*	1
Others	36
No statements	5

#### Treatment before death

The majority of children (58/100) were treated with a combination of modern and traditional methods. 25/100 (25%) were treated exclusively with modern methods and 14/100 (14%) were treated solely with traditional methods. Three children received no treatment at all. Looking at the cited local illness concepts, the majority of the *sumaya *and *kono *cases were treated with a mix of traditional and modern methods. In case of *kono*, a visit to a guérisseur was mentioned 7 times (33%). The only case of *djoliban *was also treated with a mixture of traditional and modern treatment.

#### Reasons for not visiting a modern health facility

Roughly half of the deceased children were not brought to a modern health facility during the final illness episode (n = 51). Lack of money (13/51) (25%) and a rapid progress to death (13/51) (25%) were the main reasons given to the question why no modern health facility was visited. In 17/51 (33%) questionnaires there was no specific reason stated.

## Discussion

In the light of the continuous malaria control priority on early and effective treatment, an important research focus is to investigate factors for avoidance or delay in attending modern health facilities. The decision how and where malaria is treated usually starts with the mothers' interpretation and diagnosis of their children's disease at home. The main finding from this study is that the disease perception and interpretation are crucial elements influencing treatment choices for malaria cases in rural Burkina Faso.

This study focused on the four local illness concepts s*umaya, dusukun yelema, kono*, and *djoliban*, resembling uncomplicated malaria, respiratory distress syndrome, cerebral malaria and severe anaemia respectively. However, there exist more local concepts that might overlap with the biomedical picture of malaria, as seen on Table 1.

*Sumaya *is obviously the most frequent malaria manifestation. However, *sumaya *is not spontaneously linked to mosquitoes, which supports results from other studies in Burkina Faso [21,25,26] as well as from other African countries [27-33]. Therefore, one can assume that the biomedical concept of malaria is still not yet adequately understood by the local population.

Self-treatment of uncomplicated malaria, often using a mixture of traditional and modern methods, is a common practice throughout Africa [27-35]. In the Nouna study area, treatment of childhood fever has been reported as a combination of modern and traditional methods which are generally handled by the mothers at home [2]. The findings from this study confirm such pattern of treatment behaviour which supports the potential of malaria home treatment strategies [36,37].

It can be assumed that severe malaria cases manifesting as acidosis are frequently interpreted as *dusukun yelema*, the local illness concept of the "displaced heart" with respiration difficulties as a main symptom. The possible supernatural causes (sorcerers) might be the reason that traditional guérisseurs are regarded to have the ability to treat this illness. Findings from other studies also show that in SSA people classify the causes of illness as either natural (i.e. God given) or supernatural (i.e. due to magico-religious forces) with different consequences for treatment choices [38,39]. The fact that children probably suffering from severe malaria will first be seen by a traditional healer delays prompt and effective biomedical treatment and may have fatal consequences.

The concept of *kono *(the "bird-illness") is wide-spread among the different ethnic groups in Africa. It is also called *kono *or *kononyama *among the Bambara in neighboring Mali [40], *beno*, *liula, tigaangu, jimi yie, foondu *among the Bisa, Mossi, Goin, Winyè and Fulbe ethnic groups of Burkina Faso [25,41-44] and *degedege *in Tanzania [35,45]. The findings from this study confirm that the treatment of cerebral malaria manifestations in children by local guérisseurs is a common practice in many African countries [29,32,33,35,45,46]. As in the case of *dusukun yelema*, this likely delays prompt and effective treatment and may consequently increase mortality. Such an effect is likely to be amplified by the reported belief that if a child having convulsions receives an injection, this would be fatal. Similar attitudes have also been reported from other African countries [33,35,45]. Because of such a belief in Tanzania, grandmothers and mothers in-laws strongly forbid young mothers to take a child with *degedege *to hospital [35].

The traditional treatment for *kono *is likely to have a high empirical efficacy as people are unable to distinguish malarial convulsions from the more frequent febrile convulsions. The guérisseurs thus intervene and take credit for cases that would have resolved spontaneously. In contrast, hospital treatment for cerebral malaria continues to result in a high mortality, supporting the belief that modern medicine does not cure or is inappropriate to treat convulsions [23].

The findings from the interviews show, that not much is known about *djoliban *in the study area. *Djoliban *did not exist in the local illness repertoire until the modern health services introduced this term. As trained health personnel explained and translated anaemia to the local population, the Dioula term *djoliban *("the blood is finished") was developed. The assumption that *djoliban *is no "indigenous" illness would also explain its treatment in modern health services. Winch would define *djoliban *as a "hospital disease", i.e. a disease intimately associated with and belonging to the formal health services [45].

Besides the local illness concept as a more unconscious factor influencing health-seeking behaviour and treatment choice of which the people are not directly aware, another – more obvious – important factor is lack of money that has been mentioned in the FGDs as one reason for choosing traditional treatment, which is probably influenced by the fact that traditional treatment is cheaper than the formal health care services in the study area. This is supported by similar findings from another area in rural Burkina Faso [26,47], but it is not in agreement with the findings from a study in Tanzania where traditional services were shown to be more expensive compared to modern services [48].

The information from the VA questionnaires supports the existing divergence of the biomedical malaria concept and local illness concepts [21,38] – an exact translation of the biomedical term 'malaria' into a single local term is difficult. Moreover, it provides further evidence for the often rapid fatal development of childhood diseases and particularly malaria in rural SSA [49]. Finally, it confirms the mixture of traditional and modern methods used for malaria treatment in the study area [2]. Such a mixture was also reported regarding the illness concepts *kono *and *djoliban*, whereas qualitative interviews had pointed to preferences for traditional and modern treatment respectively. But as there was no information in the VAs regarding the temporal sequence of steps taken, the reported preferences may still be valid. That treatment may change has been shown for example in Kenya, where lay people switch from one health care source to another as time passes and as their condition persists [50]. Treatment behaviour should be viewed as a process in which beliefs and actions are continuously debated and evaluated throughout the course of the illness [51].

This study also clearly shows that the guérisseurs would desire to be involved in a more active collaboration with the formal health care sector. This wish for a closer collaboration with the formal health sector was also mentioned during the course of a study on the treatment of breast pathologies associated with breastfeeding [52]. In view of the continuing important role of traditional treatment in the remote rural areas of SSA, there is an urgent need of governmental health planners to start a more open dialog with rural communities and their traditional services to comprehensively address health issues. Health education interventions should consider local terminologies and focus on improving malaria treatment seeking practices. Attitudes and beliefs that are dangerous to health should be changed by addressing the gaps in local perceptions through culturally appropriate methods.

In conclusion, this study has shown that the traditional treatment of certain manifestations of severe malaria in Africa is associated with avoidance or significant delay of effective biomedical treatment. Thus, local illness concepts need to be considered when developing specific education messages within national malaria control programs.

## Authors' contributions

CB, EW and OM designed the study. CB, AS and BK were responsible for the conduct of the study in Burkina Faso. CB, MDA and OM analysed the data. All authors contributed to the interpretation of the data, helped write the paper, and read and approved the final manuscript.

## References

[B1] Greenwood B, Bradley A, Greenwood A, Byass P, Jammeh K, Marsh K, Tulloch F, Oldfield F, Hayes R (1987). Mortality and morbidity from malaria among children in a rural area of The Gambia. Trans R Soc Trop Med Hyg.

[B2] Müller O, Traoré C, Kouyaté B, Becher H (2003). Malaria morbidity, treatment seeking behaviour, and mortality in a cohort of young children in rural Burkina Faso. Trop Med Int Health.

[B3] McCombie SC (2002). Self-treatment for malaria: the evidence and methodological issues. Health Policy Plan.

[B4] Dong H, Gbangou A, De Allegri M, Pokhrel S, Sauerborn R (2006). The differences in characteristics between health-care users and non-users: implication for introducing community-based health insurance in Burkina Faso. Eur J Health Econ.

[B5] Hamel MJ, Odhacha A, Roberts JM, Deming MS (2001). Malaria control in Bungoma District, Kenya: a survey of home treatment of children with fever, bednet use and attendance at antenatal clinics. Bull World Health Organ.

[B6] Hjortsberg C (2003). Why do the sick not utilise health care? The case of Zambia. Health Econ.

[B7] Hutton G (2004). Is the jury still out on the impact of user fees in Africa? A review of the evidence from selected countries on user fees and determinants of health service utilisation. East Afr Med J.

[B8] Kemble SK, Davis JC, Nalugwa T, Njama-Meya D, Hopkins H, Dorsey G, Staedke SG (2006). Prevention and treatment strategies used for the community management of childhood fever in Kampala, Uganda. Am J Trop Med Hyg.

[B9] Krause G, Sauerborn R (2000). Comprehensive community effectiveness of health care. A study of malaria treatment in children and adults in rural Burkina Faso. Ann Trop Paediatr.

[B10] Mugisha F, Kouyaté B, Gbangou A, Sauerborn R (2002). Examining out-of-pocket expenditure on health care in Nouna, Burkina Faso: implications for health policy. Trop Med Int Health.

[B11] Ahorlu CK, Koram KA, Ahorlu C, de Savigny D, Weiss MG (2005). Community concepts of malaria-related illness with and without convulsions in southern Ghana. Malar J.

[B12] Aikins MK, Pickering H, Greenwood BM (1994). Attitudes to malaria, traditional practices and bednets (mosquito nets) as vector control measures: a comparative study in five west African countries. J Trop Med Hyg.

[B13] Panter-Brick C, Clarke SE, Lomas H, Pinder M, Lindsay SW (2006). Culturally compelling strategies for behaviour change: a social ecology model and case study in malaria prevention. Soc Sci Med.

[B14] Tarimo DS, Minjas JN, Bygbjerg IC (2001). Perception of chloroquine efficacy and alternative treatments for uncomplicated malaria in children in a holoendemic area of Tanzania: implications for the change of treatment policy. Trop Med Int Health.

[B15] Vundule C, Mharakurwa S (1996). Knowledge, practices, and perceptions about malaria in rural communities of Zimbabwe: relevance to malaria control. Bull World Health Organ.

[B16] McCombie SC (1996). Treatment seeking for malaria: a review of recent research. Soc Sci Med.

[B17] Tanner M, Vlassoff C (1998). Treatment-seeking behaviour for malaria: a typology based on endemicity and gender. Soc Sci Med.

[B18] Sankoh OA, Ye Y, Sauerborn R, Müller O, Becher H (2001). Clustering of childhood mortality in rural Burkina Faso. Int J Epidemiol.

[B19] Müller O, Becher H, Baltussen A, Yé Y, Diallo D, Konate M, Gbangou A, Kouyaté B, Garenne M (2001). Effect of zinc supplementation on malaria morbidity among West African children: A randomized double-blind placebo-controlled trial. BMJ.

[B20] Hornung U (1998). Akute Infektionen der Atemwege bei Kindern unter fünf Jahren im kulturellen Kontext: Eine Studie in Burkina Faso.

[B21] Okrah J, Traoré C, Palé A, Sommerfeld J, Müller O (2002). Community factors associated with malaria prevention by mosquito nets: an exploratory study in rural Burkina Faso. Trop Med Int Health.

[B22] Miaffo CM (2003). Malaria and anemia prevention behavior in pregnant women in rural Burkina Faso.

[B23] WHO (2000). Severe falciparum malaria. Trans R Soc Trop Med Hyg.

[B24] Patton MQ (1980). Qualitative Evaluation and Research Methods.

[B25] Bonnet D (1986). Représentations culturelles du paludisme chez les Moose du Burkina.

[B26] Seri L (1996). Analyse du comportement des mères face à l'enfant malade du paludisme (cas du département de Gassan, province du Sourou).

[B27] Agyepong IA (1992). Malaria: ethnomedical perceptions and practice in an Adangbe farming community and implications for control. Soc Sci Med.

[B28] Abyan IM, Osman A (1993). Social and behavioural factors affecting malaria in Somalia. Social and Economic Research Project Reports no 11.

[B29] Helitzer-Allen DL, Kendall C, Wirima JJ (1993). The role of ethnographic research in malaria control: an example from Malawi. Res Sociol Health Care.

[B30] Mwenesi H, Harpham T, Snow RW (1995). Child malaria treatment practices among mothers in Kenya. Soc Sci Med.

[B31] Ruebush TK, Kern MK, Campbell CC, Oloo AJ (1995). Selftreatment of malaria in a rural area of western Kenya. Bull World Health Organ.

[B32] Ahorlu CK, Dunyo SK, Afari EA, Koram KA, Nkrumah FK (1997). Malaria-related beliefs and behaviour in southern Ghana: implications for treatment, prevention and control. Trop Med Int Health.

[B33] Nuwaha F (2002). People's perception of malaria in Mbarara, Uganda. Trop Med Int Health.

[B34] Kengeya-Kayondo JF, Seeley JA, Kajura-Bajenia E, Kabunga E, Mubiru E, Sembajja F, Mulder DW (1994). Recognition, treatment seeking behaviour and perception of cause of malaria among rural women in Uganda. Acta Trop.

[B35] Comoro C, Nsimba SED, Warsame M, Tomson G (2003). Local understanding, perceptions and reported practices of mothers/guardians and health workers on childhood malaria in a Tanzanian district – implications for malaria control. Acta Trop.

[B36] Pagnoni F, Convelbo N, Tiendrebeogo J, Cousens S, Esposito F (1997). A community-based programme to provide prompt and adequate treatment of presumptive malaria in children. Trans R Soc Trop Med Hyg.

[B37] Kidane G, Morrow RH (2000). Teaching mothers to provide home treatment of malaria in Tigray, Ethiopia: a randomised trial. Lancet.

[B38] Good CM (1987). Ethnomedical Systems in Africa.

[B39] Sommerfeld J, Sanon M, Kouyaté BA, Sauerborn R (2002). Perceptions of Risk, Vulnerability, and Disease Prevention in Rural Burkina Faso: Implications for Community-Based Health Care and Insurance. Hum Organ.

[B40] Roger M, Brunet-Jailly J (1993). Sumaya dans la région de Sikasso: une entité en évolution. Se soigner au Mali: Une contribution des sciences sociales.

[B41] Fainzang S (1986). L'intérieur des choses: maladie, divination et reproduction sociale chez les Bisa du Burkina.

[B42] Jacob JP (1987). Interprétation de la Maladie chez les Winyè, Gurunsi du Burkina Faso. Critique d'une théorie de la contamination. Genève-Afrique.

[B43] Dacher M (1988). Les représentations de la maladie chez les Goin du Burkina Faso.

[B44] Bugmann N (2000). Le concept du paludisme, l'usage et l'efficacité in vivo de trois traitements traditionnels antipalustres dans la région de Dori, Burkina Faso.

[B45] Winch PJ, Makemba AM, Kamazima SR, Lurie M, Lwihula GK, Premji Z, Minjas JN, Shiff CJ (1996). Local terminology for febrile illnesses in Bagamoyo District, Tanzania and its impact on the design of a community-based malaria control programme. Soc Sci Med.

[B46] Mwenesi HA (1994). Mothers' Definition and Treatment of Childhood Malaria on the Kenyan Coast. Social and Economic Research Project Reports no 13.

[B47] Su TT, Kouyaté B, Flessa S (2006). Catastrophic household expenditure for health care in a low-income society: a study from Nouna health district, Burkina Faso. Bull World Health Organ.

[B48] Hausmann Muela S (2000). Community understanding of malaria, and treatment-seeking behaviour, in a holoendemic area of southeastern Tanzania.

[B49] Greenwood B, Bradley A, Bypass P, Greenwood A, Menon A, Snow R, Hayes R, Hatib-N'Jie A (1990). Evaluation of a PHC programme in The Gambia. II Its impact on mortality and morbidity in young children. J Trop Med Hyg.

[B50] Nyamongo IK (2002). Health care switching behaviour of malaria patients in a Kenyan rural community. Soc Sci Med.

[B51] Oberländer L, Elverdan B (2000). Malaria in the United Republic of Tanzania: cultural considerations and health-seeking behaviour. Bull World Health Organ.

[B52] De Allegri M, Sarker M, Hofmann J, Sanon M, Boehler T (2007). A qualitative investigation into knowledge, beliefs, and practices surrounding mastitis in sub-Saharan Africa: what implications for vertical transmission of HIV?. BMC Public Health.

